# Evaluation and Comparison of the Effects of Persulfate Containing and Persulfate-free Denture Cleansers on Acrylic Resin Teeth Stained with Cigarette Smoke: An In Vitro Study

**DOI:** 10.7759/cureus.7318

**Published:** 2020-03-18

**Authors:** Meena Varshini K, Deepshika Selvakumar, Madhan Kumar Seenivasan, Shanmuganathan Natarajan, Parthasarathy Natarajan, Prathibha Saravanakumar

**Affiliations:** 1 Dentistry, Sri Ramachandra Institute of Higher Education and Research, Chennai, IND; 2 Prosthodontics, Sri Ramachandra Institute of Higher Education and Research, Chennai, IND; 3 Prosthodontics, Sri Ramachandra University, Chennai, IND

**Keywords:** denture aesthetics, denture cleansers, persulfate

## Abstract

Problem statement and aim

The esthetics of the complete denture primarily depend upon the color of the denture teeth; however, there are situations where the teeth are subjected to extrinsic and intrinsic stains and discolor over time. The purpose of the study was to investigate the effects of smoking and two different denture cleaners on the color stability of the denture teeth.

Material and methods

Commercially available maxillary anterior teeth made up of acrylic resin were selected for the study and were divided into two groups (n=10): persulfate-free denture cleanser and persulfate containing denture cleansers. The acrylic teeth were set in the smoke chamber with a distance to absorb the smoke equally from the cigarette. The smoke was released for 10 minutes, and the results are observed by the spectrophotometer.

Results

All the values were collected after the 21^st^ day, and data were analyzed with the SPSS software. It was found that denture cleansers with persulfate are effective on color stability.

Conclusions

Even though the persulfate containing denture cleansers are injurious to health, they can be recommended to the smokers with clear instructions of use. However, for non-smokers, persulfate-free denture cleansers are preferred over the persulfate containing denture cleansers.

## Introduction

Staining of acrylic dental replacement teeth has been a significant complaint from dental replacement wearers. There are a few several reasons behind denture staining, for example, long haul utilization of espresso coffee, tea, shaded beverages, and smoking [1]. The staining of resin-based materials has all the earmarks of being identified with numerous elements and might be brought about by natural and outward factors. The characteristic elements include the staining of the resin material itself; for example, the change of the resin matrix by physicochemical responses causing disintegration [2]. Since high-quality plastic teeth made of hard resin are helpless to staining with colors, it has been seen that the esthetics of removable partial prosthesis made using such plastic teeth slowly decrease in numerous patients. Then again, porcelain teeth are nearly predominant as a result of their high wear opposition, less perception of stains, and have a great esthetical appearance. Nonetheless, if removable partial prosthesis were utilized for significant use without adequate cleaning, there is a chance of staining in porcelain teeth too due to the adsorption of colorants on the porcelain surface [3]. Consequently, dental specialists suggest dental replacement chemicals for the cleaning of the dentures, which have persulfate as the major component and persulfate-free denture cleansers [4]. In respect to this, the aim of the study is to discover the effectiveness of the persulfate-free dental replacement chemicals when exposed to smoke.

## Materials and methods

**Figure 1:**
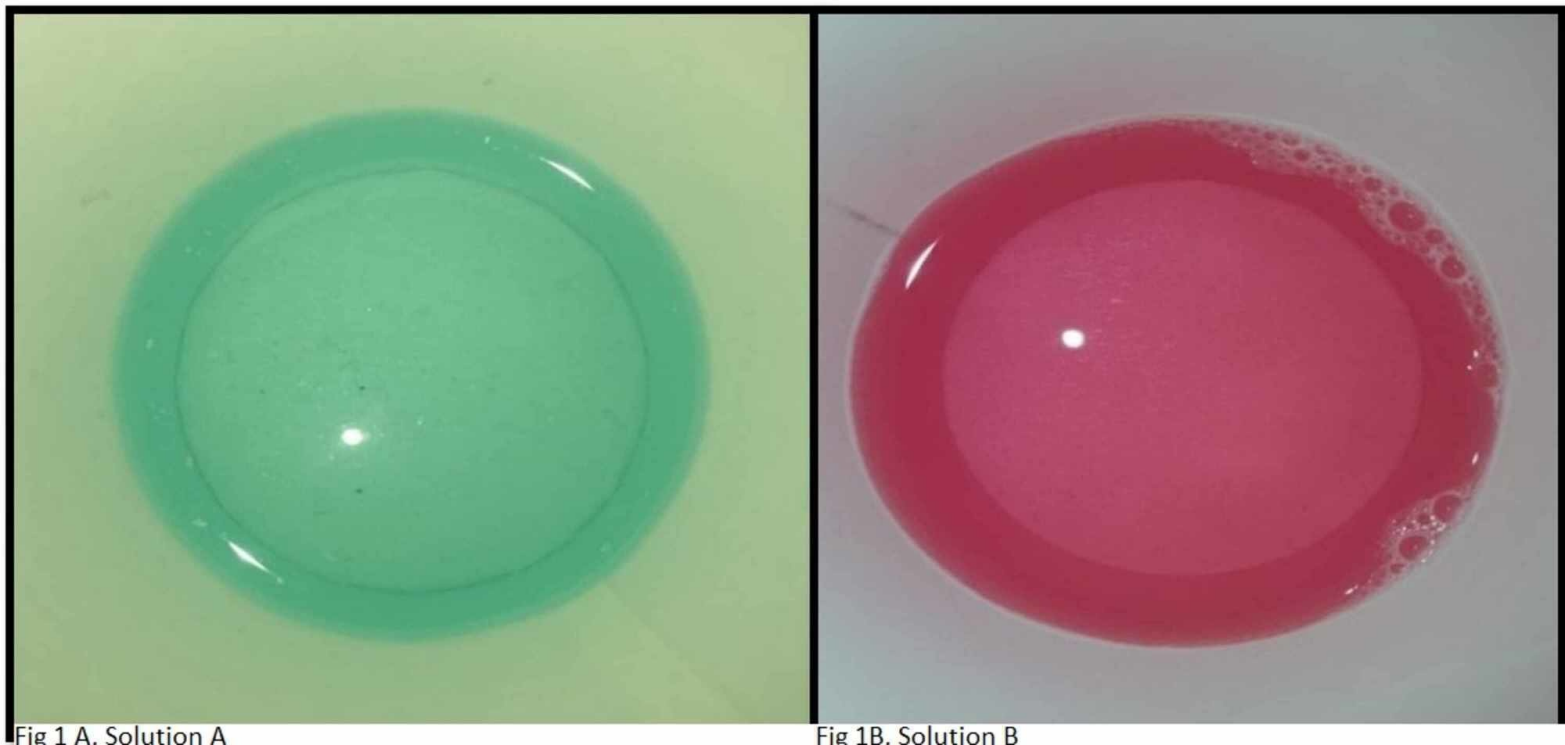
Denture cleanser without persulfate (solution A) and denture cleanser with persulfate (solution B)

Twenty sets of maxillary anterior resin teeth were selected for the study and were grouped into group A and group B, with each group having 10 sets each. Two denture cleansers were used for this study - one without persulfate (Dentasoak®; solution A) and other with persulfate (Polident®; solution B). Solution A (Dentasoak®) was prepared by blending the powder and solution in 50ml of distilled water; solution B (Polident®) was prepared by diluting one tablet in 50ml of distilled water as per the manufacturer’s instructions (Figure 1 A and B).

Construction of smoke chamber

**Figure 2:**
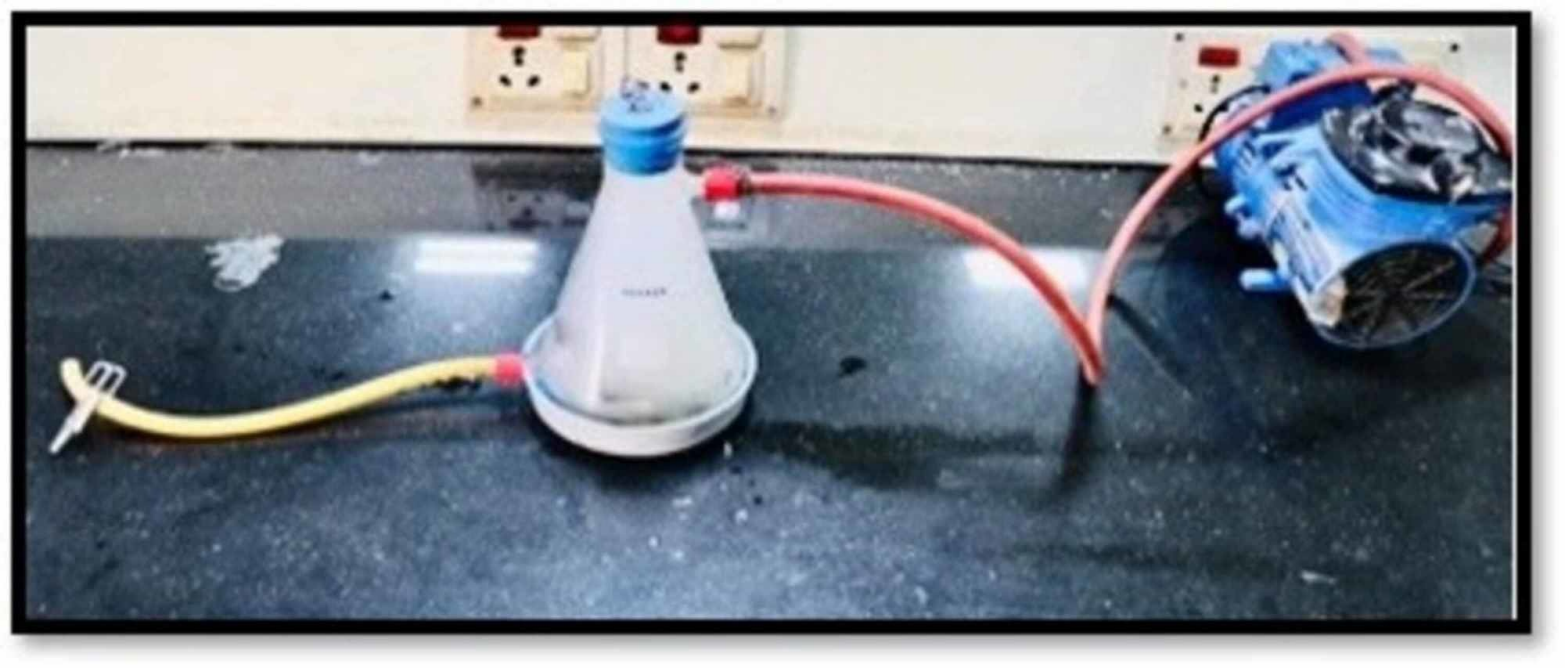
Custom made smoke chamber

**Figure 3:**
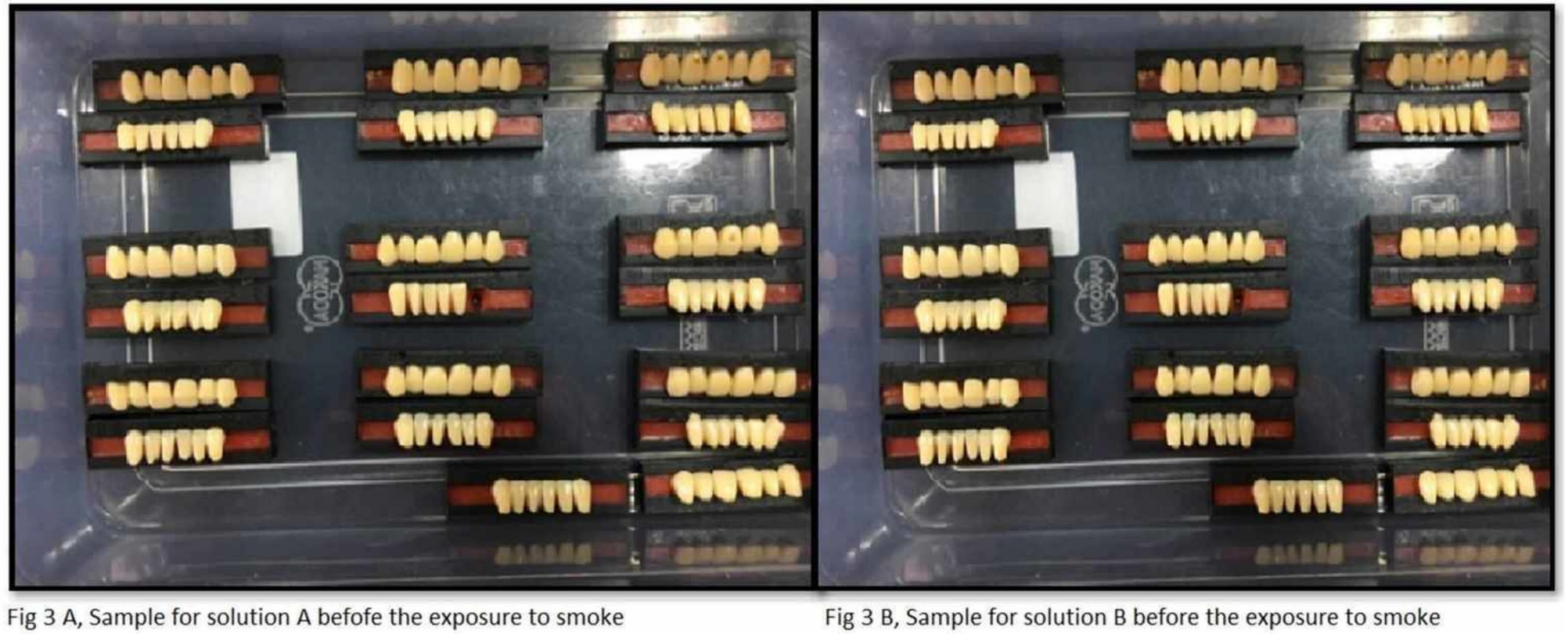
Samples after exposure to smoke and before exposure to denture cleanser on day 1 (groups A and B)

**Figure 4:**
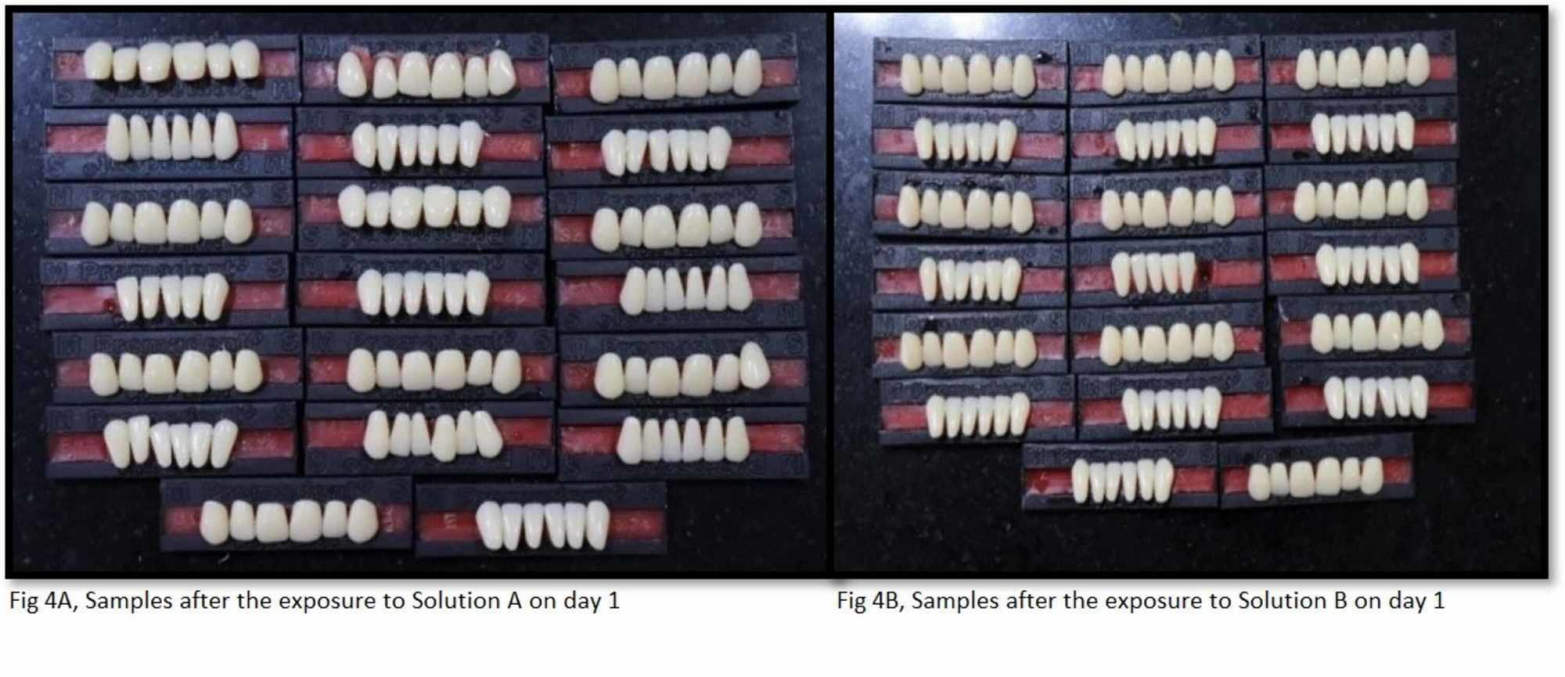
Samples after the exposure to denture cleanser on day 1 (group A, solution A and group B, solution B)

**Figure 5:**
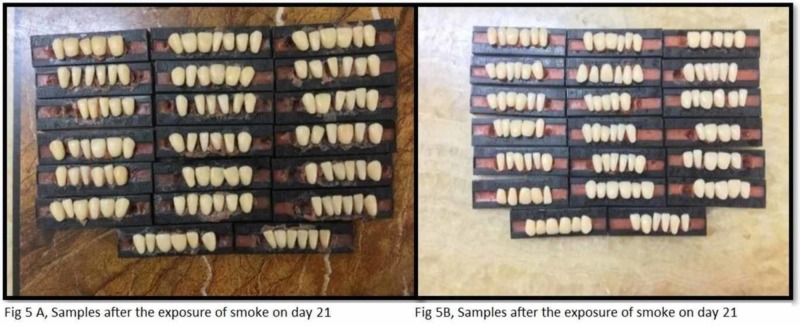
Samples after exposure to smoke on day 21 (groups A and B)

**Figure 6:**
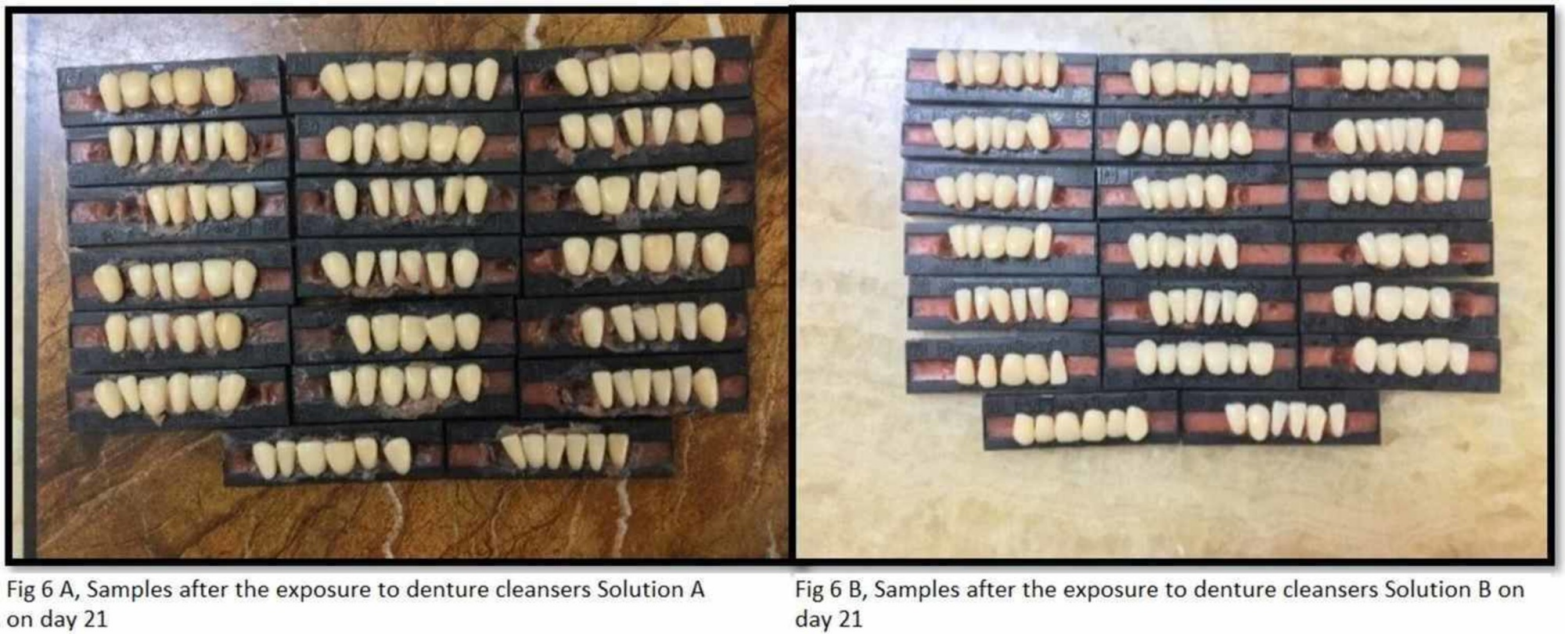
Samples after exposure to denture cleanser on day 21 (group A, solution A and group B, solution B)

The study utilized the smoking machine created by the standards of Wasilewski S et al. to recreate (in vitro) the smokers' mouth conditions [2]. The smoke chamber is utilized to expose the tobacco smoke to the acrylic teeth. It has a glass beaker with two openings that are linked with silicone tubes (Figure 2). One silicone tube is connected to the vacuum and the other tube is locked. The conical glass beaker is closed with a stopper (cork) and has a hole in the center; this hole is utilized to embed the cigarette. At the point when the vacuum is actuated, the tobacco smoke is discharged into the container because of negative pressure from the vacuum. Acrylic teeth were assembled in such a manner that the labial surface of the teeth was confronting the inlet to ingest the smoke when it is discharged from the opening. For equivalent retention of the smoke, all the samples were kept and balanced out at an equivalent separation. At that point, the acrylic dental replacement teeth are along these lines presented to tobacco smoke for 10 minutes. Each group (group A and group B) acrylic resin teeth are exposed to tobacco smoke independently five times each day for 10 minutes. The exposure of the smoke is completed five times each day since it's been anticipated that a normal smoker smokes at least five cigarettes every day. Figures 3 A and B show the samples after exposure to smoke and before exposure to denture cleanser on day 1 (groups A and B). Figures 4 A and B show the samples after the exposure of denture cleansers on day 1. Figures 5 A and B show the samples after the exposure of the smoke on day 21, and Figures 6 A and B represent the aftereffect on the acrylic teeth after subjecting to denture cleansers on day 21. Later the group A teeth are drenched into solution A, and group B teeth are exposed to solution B for overnight. All the samples were collected after 21 days and tested in a spectrophotometer for color intensity.

Spectrometric analysis of color intensity

**Figure 7:**
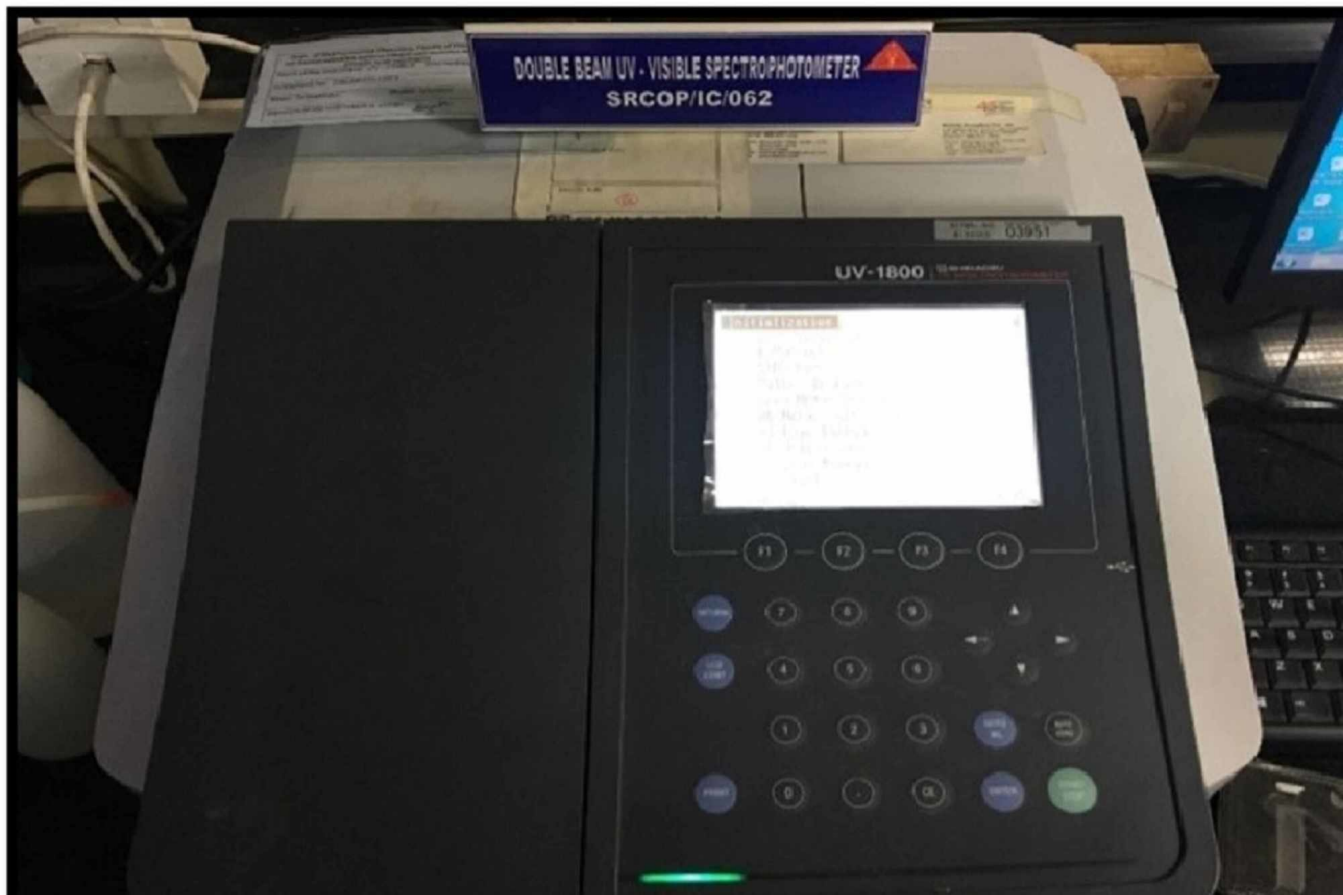
Double-beam UV-visible spectrophotometer

We utilized a double-beam UV-visible spectrophotometer (Figure 7). Color changes can be assessed outwardly or by instrumental techniques. Instrumental valuations eliminate the subjective interpretation of visual coloring examination [5]. This spectrophotometer utilizes a reference beam and sample beam shaft that go through the sample solutions. The two denture cleansers absorb the various wavelengths of light. The measure of absorption is reliant on the convergence of the denture cleansers. The qualities recorded from the arrangements allude to the shading force of the arrangement. The acrylic teeth were presented to tobacco smoke for 21 days and were absorbed dental replacement chemical medium-term for 21 days. The color intensity values are recorded on first, third, fifth, seventh, ninth, 11th, 13th, 15th, and 21st days. Prior to every measurement session, the spectrophotometer was calibrated according to the manufacture’s recommendations to avoid the wrong measurement.

The materials used in the study are described in Table 1. 

**Table 1:** Materials used in the study

Material	Brand name	Composition
Acrylic resin teeth	Premadent®	Polymethyl methacrylate, benzoyl peroxide, methyl methacrylate, ethylene glycol dimethacrylate, hydroquinone N’N dimethyl p-toluidine
Denture cleanser (solution A)	Dentasoak®	Sodium, bicarbonate, citric acid, sodium perborate, mint flavor, sodium sulfate, ethylenediaminetetraacetic acid
Denture cleanser (solution B)	Polident®	Sodium persulfate, sodium hypochlorite, sodium bicarbonate, sodium perborate, sodium polyphosphate, ethylenediaminetetraacetic acid
Smoke chamber	Custom made	
Spectrophotometer	Double-beam UV	

## Results

The spectrophotometric values on the exposure of the denture cleansers were observed (from day 1 to day 21) and tabulated in Table 2. These values are then subjected to statistical analysis with SPSS software 24 (IBM Inc., Armonk, USA). In the present study, two separate investigations were performed to compare the values corresponding to ∆E. Spectrophotometric values of group A and group B were compared by using an independent t-test. Table 2 shows that there is a significant difference between two groups (p-value < 0.001). The results of the photographic examination revealed a significant difference between group A and group B denture cleansers. Persulfate containing denture cleanser (group B) is more effective than persulfate-free denture cleanser (group A) in removing the cigarette smoke stain in acrylic resin teeth.

**Table 2:** The color intensity values refers to amount of smoking stains absorbed by the denture cleanser

Days	Group A	Group B
1	0.118	0.210
3	0.210	0.148
5	0.335	0.121
7	0.395	0.144
9	0.310	0.108
11	0.285	0.098
13	0.203	0.037
15	0.190	0.067
17	0.158	0.082
21	0.126	0.099

## Discussion

Denture cleansers control the development of microorganisms on false teeth, remove stains and different flotsam and jetsam brought by diet, tobacco, smoking, espresso, and so on. A few denture cleansers come in cream and fluid forms; others come in powders, glue, or tablet forms. Commercial denture cleansers are classified into the following groups: unbiased peroxides with compounds, proteins, acids, hypochlorite's, peroxides, rough medications, and mouthwashes for dentures [6]. The decision of denture cleansers should be based on the chemistry of resin and cleanser, denture cleanser concentration, and duration of immersion. Regular utilization of denture cleansers is prescribed to prevent microbial colonization on the dentures and promote great oral wellbeing. Daily utilization of denture cleansers can influence the physical and mechanical properties of denture base material [7]. Most generally utilized denture cleansers are represented by the group of alkaline peroxides. Potassium monopersulfate removes staining and eliminate microbes on a denture surface [8]. Considering the cleaning strategies utilized by the patients on the dentures, resin materials were drenched in 50-degree centigrade warm solutions for 15 minutes every day for 20 days.

Seventy-three reports of susceptible allergic responses, including one demise connected to denture cleanser, had been accounted for by the FDA (Food and Drug Administration) referring to persulfate as a reason [9]. Side effects of unfavorably susceptible responses to persulfates utilized in denture cleanser are bothering, tissue harm, rash, gum delicacy, breathing issues, low circulatory strain, and overall hypersensitive response. The outcomes showed that there were contrasts in the stain removal effectiveness of both the denture cleanser. Looking at two denture cleansers, we came to the outcome that the persulfate containing dental replacement chemical is progressively more viable in expelling the cigarette smoking stains from the dental replacement tar acrylic teeth than the persulfate-free dental replacement chemical. Considering the wellbeing variable of the patient's persulfate-free dental replacement chemical, it can be prescribed for non-smoker patients. For smokers, the persulfate containing dental replacement chemicals ought to be suggested yet with appropriate safety measures and directions to stay away from any complications. Within the restrictions of this investigation, we conclude that the most proficient dental replacement cleaning agents in expelling tobacco smoke stains are persulfate containing dental replacement cleaning agents. Further examinations with more samples and a more extended range of the investigations in vivo will give more exact results. In addition, checking the physical properties like the quality of the acrylic teeth after the introduction of the dental replacement chemicals could add to the study.

## Conclusions

FDA warns of allergic reactions to persulfate containing denture cleansers with proper or improper use. The importance of persulfate-free denture cleansers has to be acknowledged. In the case of non-smokers, the use of persulfate-free denture cleansers should be encouraged for safety concerns. In the case of smokers and others who use persulfate containing denture cleansers, it is important to make sure they follow the instructions and use it with precautions.
